# Phenolic Composition and Biological Properties of *Rhus microphylla* and *Myrtillocactus geometrizans* Fruit Extracts

**DOI:** 10.3390/plants10102010

**Published:** 2021-09-25

**Authors:** Jorge L. Guía-García, Ana V. Charles-Rodríguez, Julio C. López-Romero, Heriberto Torres-Moreno, Zlatina Genisheva, Armando Robledo-Olivo, M. Humberto Reyes-Valdés, Francisca Ramírez-Godina, Hermila T. García-Osuna, María L. Flores-López

**Affiliations:** 1 Universidad Autónoma Agraria Antonio Narro, Calzada Antonio Narro No. 1923, Colonia Buenavista, Saltillo 25315, Mexico; jorge.guia.g92@gmail.com (J.L.G.-G.); armando.robledo@outlook.com (A.R.-O.); mathgenome@gmail.com (M.H.R.-V.); godramf@gmail.com (F.R.-G.); hgosuna@hotmail.com (H.T.G.-O.); 2 Departamento de Ciencias Químico Biológicas y Agropecuarias, Universidad de Sonora, Unidad Regional Norte, Ave. Universidad e Irigoyen, H. Carboca 83600, Mexico; julio.lopez@unison.mx (J.C.L.-R.); heriberto.torres@unison.mx (H.T.-M.); 3 CEB—Centre of Biological Engineering, University of Minho, Campus de Gualtar, 4710-057 Braga, Portugal; zgenisheva@gmail.com; 4 Universidad Interserrana del Estado de Puebla Ahuactlán, Ahuacatlán 73330, Mexico

**Keywords:** *Rhus microphylla*, *Myrtillocactus geometrizans*, ohmic heating, phenolic compounds, antioxidant activity, antiproliferative activity, antifungal activity

## Abstract

Plants from arid zones of Mexico are an interesting source of phytochemicals that exhibit a large number of biological properties. In this context, *Rhus microphylla* (Rm) and *Myrtillocactus geometrizans* (Mg) fruits have been used as folk remedies and to make traditional foods, respectively; however, studies on their composition and bioactivity are limited. Thus, the objective of this work was to evaluate the yields, phenolic composition, and bioactive properties (scavenging and reducing capacities, antiproliferative, and antifungal) of aqueous and hydroalcohol extracts of Rm and Mg fruits obtained by conventional agitation and ohmic heating (OH). The results showed that the Rm fruit extracts had the highest total phenolic content (TPC) values and the strongest scavenging and reducing capacities compared to those of Mg fruits, being characterized by the presence of gallic acid, while the composition of the Mg extracts varied with respect to the extraction conditions used. Regarding antifungal activity in vitro against two phytopathogenic fungi, *Rhizopus stolonifer* and *Fusarium oxysporum*, the hydroalcohol extracts obtained by conventional agitation of both plants (RmH-C and MgH-C) showed the best inhibitory effect, respectively. Interestingly, none of the extracts under study presented cytotoxicity against the noncancerous ARPE-19 cell line, while three extracts of Rm fruit exhibited a moderate antiproliferative activity against HeLa (cancerous) cell line. These findings reveal for the first time the potential of Rm and Mg fruits as a new source of bioactive compounds for future industrial applications.

## 1. Introduction

Bioactive compounds (BCs) are produced by the secondary metabolism of plants, mainly as part of their defense system, as a barrier against pathogen agents and ﻿extreme climatic conditions [1]. Depending on their nature (e.g., phenolic compounds, anthocyanins, carotenoids, among others), the BCs can have different functions in the plant [2,3,4]. Humans have empirically used these compounds by preparing herbal infusions for treatment of dysentery, fever, diarrhea, stomach aches, and general ailments [5]. Recent studies have been directed to elucidate the potentialities of the BCs, especially as therapeutic agents in pharmaceutical and cosmetic industries [6], and as preservatives, colorants, fertilizers, and antimicrobials in the agrifood industry [7].

There are different techniques to obtain BCs, varying in the use of solvents, operating times, and temperatures [8]; and these can be divided into two types: conventional and nonconventional [9]. The first includes the most common and simple techniques, such as infusion, hydrodistillation, Soxhlet, and agitation, which have been used successfully over the years [10,11]. However, they are characterized by requiring large quantities of harmful solvents (e.g., methanol, ether, hexane, etc.) and usually long operating times, increasing the energy consumption [11,12]. Nonconventional techniques have been developed to overcome these drawbacks, among which are ohmic heating (OH), ultrasound, microwaves, and supercritical fluids [13,14,15]. These require lower operating times, less solvent use, in addition to allowing higher yields with better properties of the extracted BCs [15,16].

On the other hand, Mexico has a great diversity of plants, highlighting those that grow in arid areas due to their metabolic machinery [2]. Among these, wild species of *Myrtillocactus geometrizans* (Mart. ex Pfieff.) Console (known as garambullo) (Mg) and *Rhus microphylla* Engelm. (known as agrito) (Rm), develop interesting fruits composed mainly of flavonoids, phenolics acids, betacyanins, and betaxanthins [17,18]. The fruits vary in size and color, while Mg fruits are juicy, spheric, and purple, Rm fruits are small, spheric, orange, and dry drupe [19,20]. Mg fruit has been reported for its high antioxidant [1] and antihyperglycemic [21] activities, while the methanol extracts from roots and aerial parts of Mg have shown an insecticidal effect on the fall armyworm (*Spodoptera frugiperda*) [22]. Regarding Rm fruits, their ethanol and aqueous extracts were recently reported for their strong antifungal activity in vitro against two important crop pathogens, *Fusarium oxysporum* and *Corynespora cassiicola* [17]. The application of novel green extraction technologies, such as OH, represents an excellent tool for the recovery of crude extracts and BCs from these species. The OH extraction involves an electric field to promote the depolarization of the cellular wall and the consequent release of BCs from the matrix, being critical to study several parameters to optimize its performance (i.e., voltage, operational times, solute: solvent ratio, temperature, and conductivity) [23]. The OH has allowed better yields (13.2%) of clove essential oil than those with simple hydrodistillation (8.2%) [15]. In addition, the yields of hydroalcohol *Pinus pinaster* bark extracts have shown an increase of 30% when compared to conventional heating [23].

Despite the promising potential of Rm and Mg fruits, research on the possible benefits of extracts from both plants in different industrial areas is still scarce. Therefore, the objective of this work was to determine the yields and phenolic composition of aqueous and hydroalcohol extracts of *R. microphylla* and *M. geometrizans* fruits obtained by means of conventional agitation and OH; additionally, their bioactive properties as antioxidant, antiproliferative, and antifungal agents were determined.

## 2. Results

### 2.1. Yields and Total Phenolic Content of Extracts

Table 1 shows the extract yields and TPC values for Rm and Mg fruits obtained by conventional agitation and OH technique. The maximum yields (*p* < 0.05) for Rm fruits were obtained by conventional agitation, in addition these conditions allowed a higher recovery of TPC with values of 75.34 ± 6.48 and 62.00 ± 3.34 mg GA/g extract using aqueous and hydroalcohol solution as extracting agents, respectively. Significantly lower yields were observed with the application of OH, but when it was combined with hydroalcohol solution and extraction time of 10 min (RmH-OH10) presented a noteworthy value of TPC (41.37 ± 4.25 mg GA/g extract).

Regarding the Mg fruits, the yields of the extracts obtained by conventional agitation did not show significant differences according to the solvent used, being in the range of 37.63 to 43.96%. The OH extracts yields were not influenced (*p* > 0.05) by the extraction conditions, only the extract MgA-OH10 stood out as it showed values similar to those obtained with conventional agitation (40.07 ± 1.28%). However, the extraction technique and conditions did not affect the recovery of TPC, which were lower than those detected in Rm fruits.

Furthermore, the experimental variables, yields and TPC, were correlated following Equation (1) for OH extraction, and the resulting equations that describe the variation in the responses and the *R*^2^ value of each model are shown in Table 2. The *R*^2^ values were between 0.74 and 0.97, which shows that the models used might be suitable for future estimations of the factors analyzed.

### 2.2. Phenolic Profile by UHPLC

The phenolic profile was identified by means of UHPLC (Table 3 and Table 4). In general, a total of six and five different phenolic compounds were identified in the Rm and Mg fruit extracts, respectively. Gallic acid was the most abundant compound in all Rm fruit extracts, mainly in those obtained by conventional agitation (RmA-C and RmH-C), where the aqueous solvent significantly influenced in the content of this compound (*p* < 0.05). For the Mg fruit extracts, the solvent and extraction technique used also influenced the yields and the presence of phenolic compounds; the occurrence of rosmarinic acid (12.36 ± 0.01 mg/L) in the MgH-C extracts (Table 4) was particularly notable.

### 2.3. Biological Activity of Extracts

#### 2.3.1. Scavenging and Reducing Properties

The relation between the antioxidant capacity of the plant extracts and their bioactivity is well known. Thus, this parameter was determined through the radical scavenging activity (RSA) measured by the DPPH and ABTS assays, as well as the ferric reducing power by the FRAP assay (Table 5). In general, the Rm fruit extracts evidenced a stronger scavenging and reducing capacities compared to those of Mg fruits in all assays. The Rm fruit extracts obtained by conventional agitation with both solvents had higher DPPH radical scavenging activity and higher ferric reducing power compared with the OH extracts (*p* < 0.05); meanwhile for the ABTS results, only the aqueous extracts showed differences (*p* < 0.05) as a function of the extraction technique employed. Furthermore, the results observed in the DPPH and FRAP assays for the OH aqueous extracts (RmA-OH5 and RmA-OH10) were significantly influenced by the operating times (*p* < 0.05). Otherwise, in the EC_50_ values obtained in ABTS assay, the operating times did not affect (*p* > 0.05) the results of scavenging capacity in OH extracts.

On the other hand, the hydroalcohol extracts of Mg fruits showed similar behavior to that of hydroalcohol extracts of Rm fruits in the three assays evaluated, highlighting the stronger capacity of the MgH-C, with EC_50_ values of 5.25 and 3.79 mg/mL for DPPH and ABTS assays, respectively, and a higher ferric reducing power (255.78 µM Fe (II)/g extract). Furthermore, the operating times significantly affected the EC_50_ values of the OH aqueous extracts (MgA-OH5 and MgA-OH10) examined with the DPPH assay; for the OH hydroalcohol extracts, minor extraction time (MgH-OH5) allowed better reducing capacity of Fe^3+^ in comparison with its counterpart with a longer extraction time (MgH-OH10).

#### 2.3.2. Cell Viability Assay

The cytotoxicity of the extracts against cell lines was studied through the cell viability assays. The twelve extracts showed no cytotoxicity against the noncancerous cell line (ARPE-19), even at high concentrations (>800 µg/mL) (Table 6). Regarding the antiproliferative activity against HeLa (cancerous cell line), only the hydroalcohol extracts of Rm fruits (RmH-C, RmH-OH5, and RmH-OH10) showed moderate effectiveness, highlighting the RmH-C extract as it showed significant effect with an IC_50_ value of 417.73 ± 29.06 µg/mL. These results propose for the first time the antiproliferative capacity of the Rm fruit extracts, which could be selective according to the extraction conditions.

#### 2.3.3. Antifungal Activity In Vitro

**Figure 1:**
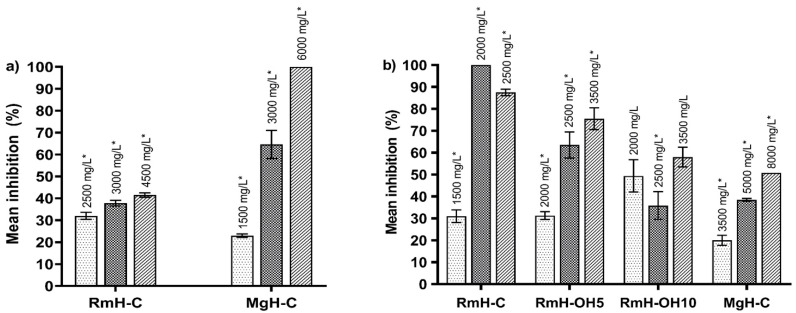
Mean inhibition (%) of *R. microphylla* (Rm) and *M. geometrizans* (Mg) fruit extracts against *F. oxysporum* (**a**) and *R. stolonifer* (**b**). Values are expressed as mean ± standard deviation (error bars), *n* = 4. The asterisk indicates significant difference between concentrations in each extract (*p* < 0.05). H: hydroalcohol extract; C: conventional agitation; OH: ohmic heating; 5 and 10: correspond to operating time (min) for OH extraction.

Figure 1 shows the Rm and Mg fruit extracts with the highest antifungal effect on *F. oxysporum* and *R. stolonifer*. The MgH-C extract was more effective in inhibiting the growth of *F. oxysporum*, showing 100% inhibition at 6000 mg/L, whereas, the RmH-C extract did not exceed 50%. Regarding *R. stolonifer*, the RmH-C extract presented complete inhibition at 2000 mg/L; additionally, good inhibitions (in the range of 50–75%) were achieved at doses of 3000, 3500, and 8000 mg/L for the RmH-OH5, RMH-OH10, and MgH-C extracts, respectively. It is important to note that only some extracts (MgH-C, RmH-OH5, and MgH-C) showed a concentration-dependent effect, which is interesting for its application as antifungal agents.

The ability of Rm and Mg fruit extracts to inhibit the growth of *R. stolonifer* and *F. oxysporum* is also reported as the minimum extract concentration required to inhibit 50 and 90% of the mycelia growth of fungi (MIC_50_ and MIC_90_, respectively) (Table 7). The RmH-C extract showed the higher antifungal activity on *R. stolonifer*, as lower values of MICs were observed (1599 and 2219 mg/L for MIC_50_ and MIC_90_, respectively). In addition, the Rm extracts obtained by means of OH showed MIC_50_ values in a range of 2366–2918 mg/L, whilst the MIC_50_ value for the MgH-C extract was approximately three-fold higher (8415 mg/L) for the inhibition of *R. stolonifer*. Otherwise, the MgH-C extract evidenced stronger antifungal activity against *F. oxysporum*, with MIC_50_ and MIC_90_ values of 1915 and 4881 mg/L, respectively. The other extracts only inhibited mycelia growth of *F. oxysporum* at higher concentrations (up to 5940 mg/L). These results exhibit the specificity of some Rm and Mg fruit extracts to inhibit the development of the fungi under study.

## 3. Discussion

In the present work, two extraction techniques (conventional agitation and OH) were evaluated on the yields, phenolic composition, and bioactive properties of Rm and Mg fruit extracts. In the OH extraction, the conductivity of the water provides a higher conductivity allowing a better dispersion of the electric current and promoting an increase in permeability within the cell wall with the subsequent appearance of pores through which the solvent enters and releases the BCs to the medium, thus increasing the extraction yields [23].

On the other hand, the highest yields obtained for RmH-C can be associated with the fact that conventional agitation is a gentler procedure that in combination with hydroalcohol solution as solvent, allowed a greater release of free phenolic compounds located inside of the cell vacuoles; in addition, the solubility of the phenolic compounds is improved owing to the solvent employed [24]. Otherwise, the OH technique can be helpful to enhance the extraction of intracellular BCs from plants (e.g., flavonoids, anthocyanins, etc.) because the applied electrical current causes a partial or total rupture of the membrane by the electroporation generated and the consequent increase in temperature [16,23]. These results agreed with the TPC values and phenolic composition, as those extracts obtained by conventional agitation and a hydroalcohol solution as an extracting agent for both plants (RmH-C and MgH-C) were those that showed the best performance for these parameters. Generally, the Rm fruit extracts obtained by conventional agitation were characterized by the presence of gallic acid and p-coumaric acid+epicatechin as main compounds, which probably affected their strong DPPH and ABTS scavenging capacities, as minor values of EC_50_ indicate higher activity [25]. Recently, extracts of *Ephedra alata* containing these phenolic compounds were reported by Benabderrahim [26] as powerful antioxidant agents. It has been reported that the antioxidant capacity of phenolic compounds results from two mechanisms, by the donating a hydrogen atom or by acting as electron donors; additionally, there is a close relationship between the structure and the antioxidant capacity of these compounds, which is associated with the presence and number of hydroxyl groups [27]. It was also noted that according to the classification reported by Wong [28], five Rm fruit extracts can be classified as strong reducing agents (RmA-C, RmA-OH5, RmA-OH10, RmH-C, and RmH-OH5), as they presented a high ferric reducing power with values of >500 µm Fe (II)/g per extract. The lower scavenging and reducing capacities of Mg fruit extracts are consistent with their lower TPC values and their minor phenolic composition [29]. Several BCs, such as epigallocatechin, protocatechuic acid, rutin, kaempferol, among others, have been reported in other plants of the *Rhus* genus [30,31]. Recently, Montiel-Sanchez [21] reported the presence of betaxanthins such as indicaxanthin and vulgaxanthin I; betacyanins, mostly phyllocactin and betanin; and some other phenolic compounds such as rutin and quercetin derivatives in the pulp, skin, and whole fruit of *M. geometrizans*.

Some of these phenolic compounds detected in Rm and Mg fruit extracts (e.g., gallic acid, *p*-coumaric acid, and ferulic acid) have been reported for their cytotoxicity properties against cancerous cell line cultures, such as in human breast cancer MCF-7 cell line, human prostate cancer cells (PC-3), and lung cancer cells (A549) [32,33]. The anticancer activity of these compounds is caused by the promotion and generation of reactive oxygen species and the arrest of the cell cycle, which induces apoptosis; furthermore, the synergism between compounds can improve their bioactivities [34,35]. López-Romero [36] identified the presence of epicatechin in extracts of *Litsea glaucescens*, having antiproliferative effect against HeLa cancer cell line (IC_50_ of 45.80 µg/mL) and selectivity between cancerous and noncancerous cell lines. Similarly, Rm and Mg fruit extracts did not show cytotoxicity against the noncancerous ARPE-19 cell line; in addition, three extracts showed selectivity (RmH-C, RmH-OH5, and RmH-OH10) by presenting moderate activity against HeLa. However, they cannot be considered as cytotoxic extracts, according to the US National Cancer Institute, as only extracts with an IC_50_ value of < 30 µg/mL can fit into this classification [37]. It is important to note that this is the first report of the cytotoxicity of extracts from Rm and Mg fruits against ARPE-19 and HeLa cell lines.

One of the most important effects reported for plant extracts is their ability to inhibit the mycelial development of fungi, which leads to their potential use as biofungicides [38]. In this context, RmH-C extract stood out for its potent antifungal activity against *R. stolonifer*, an important phytopathogenic fungus. This extract showed growth inhibition at a lower concentration (MIC_50_: 1599 mg/L) than those reported by Yang and Jiang [39] for tea polyphenols (mainly containing catechins) with MIC_50_ values of 2900 mg/L. For *F. oxysporum* (a very devastating crop pathogen), the MgH-C extract achieved lower MIC_50_ values (1915 mg/L) than those reported by Jasso de Rodríguez [40] for ethanol extracts of *R. muelleri* (MIC_50_ of 3363 mg/L). These results can be attributed to the interaction of phenolic acids and flavonoids present in the plant extract matrix of the Mg and Rm fruits, because although the mechanism of action of the extracts to inhibit mycelial growth is not completely elucidated, it is known that phenolic compounds can act against pathogens through enzymatic inhibition by oxidation of the fungal cell membrane [2,41,42].

Generally, the technique and conditions used can impact the nature and quantity of phenolic compounds isolated from the plants under study. Considering the demonstrated biological activities of Mg and Rm fruit extracts, they can be a novel proposal for the development of biofungicides, being an interesting alternative for synthetic products that affect the environment and health. In addition, their low cytotoxicity results indicate that they can be incorporated as a natural source of BCs in foods, having a positive impact on their properties.

This work provides the basis for future research, where the antiproliferative and antifungal potential of Mg and Rm fruit extracts is evaluated against other cell lines and fungal strains, respectively, in addition to the elucidation of the other compounds associated with their bioactivity.

## 4. Materials and Methods

### 4.1. Materials and Strain

Sodium carbonate (Na_2_CO_3_), gallic acid (GA), potassium persulfate (PP), 2,2-diphenyl-1-picrylhydrazil (DPPH), 2,2-azinobis-(3-ethylbenzothiazoline-6-sulphonic acid) (ABTS), 3-(4,5-dimethylthiazol-2-yl)-2,5-diphenyltetrazolium bromide (MTT), 6-hydroxy-2,5,7,8-tetramethylchroman-2-carboxylic acid (Trolox), 2,4,6-tri(2-pyridyl)-striazine (TPTZ), iron (III) chloride hexa-hydrate, dimethyl sulfoxide (DMSO), and all standards reagents for UHPLC analysis were purchased from Sigma (Sigma-Aldrich, Saint Louis, MO, USA). Absolute ethanol (99.9%) was obtained from Jalmek (Jalmek Científica S.A. de C.V., San Nicolás, NL, Mexico). Potato dextrose broth (PDB) was purchased from TM MEDIA (Titan Biotech Ltd., Delhi, India). The Folin–Ciocalteu (FC) reagent was from Merck (Merck KGaA, Darmstadt, Germany). All standards, samples, and eluents were prepared using Milli-Q water (Millipore, Bedford, MA, USA).

*Rhizopus stolonifer* (CDBB accession no. 1384) was supplied from CINVESTAV (Centro de Investigación y de Estudios Avanzados del IPN, CDMX, Mexico), and *Fusarium oxysporum* (National Center for Biotechnology Information, NCBI, accession no. MT001892) was acquired from CICY (Yucatan Center for Scientific Research, Yucatan, Mexico).

HeLa (human cervix carcinoma) and ARPE-19 (human retinal pigmented epithelium) cell line cultures were obtained from the American Type Culture Collection (ATCC, Rockville, MD, USA). Cells were cultured in Dulbecco’s Modified Eagle Medium (DMEM) supplemented with 5% fetal bovine serum (FBS) (Sigma-Aldrich, Saint Louis, MO, USA).

### 4.2. Plant Material

Rm fruits were randomly collected in wild areas located in the city of Saltillo, in Coahuila State, Mexico (25°20′44.5′’N 101°01′48.7′’W), and Mg fruits were collected in Pozo Hondo, Guanajuato State, Mexico (21°24′33.9′’N 100°36′27.8′’W) from April to May 2019. The samples were transported in plastic bags to the Fermentation Laboratory at the Universidad Autónoma Agraria Antonio Narro (UAAAN); the collected fruits were washed with distilled water and dried in a stove (Biobase Biodustry Shandong Co., Ltd., Jinan, SHG, China) at 60 °C for 48 h [38]. Subsequently, the fruits were ground to obtain a particle size equivalent to mesh no. 20; then the samples were stored in bags in a dark place until further use.

### 4.3. Preparation of R. microphylla (Rm) and M. geometrizans (Mg) Fruit Extracts

#### 4.3.1. Extraction by Ohmic Heating (OH)

The laboratory-built OH system consisted of a power supply (Voltage Autotransformer, NAPEE, Mexico), two iron electrodes, a circulating water bath, and a three-neck flask. The extraction was conducted by evaluating two parameters (solvent and operational times) using a 2^2^ factorial design (Table 8). For the experiment, a dried sample (20 g) was placed in the three-neck flask containing 400 mL of solvent, water, and a hydroalcohol solution (50:50), the electrodes were placed in the flask applying a voltage of 70 V in all of the experiments (conditions were chosen from a preliminary study, data not shown), and the temperatures were maintained below the boiling points of the solvents (99 °C for water and 75 °C for the hydroalcohol solution, respectively). The extracts obtained were filtered using a vacuum pump, and subsequently concentrated using a rotary evaporator (IKA RV 10 basic, IKA Werke GmbH and Co, KG, Staufen, Germany). Finally, the extracts were stored in the dark at 5 °C until further analyses were performed.

The experimental data (yields and TPC) for extracts obtained by means of OH extraction were analyzed with a linear first-order regression model with the following general regression equation:

(1) $$y=\beta_{0}+\beta_{1}x_{1}+\beta_{2}x_{2}+\beta_{12}x_{1}x_{2}$$

where *y* is the response (dependent variable); *β*_0_, *β*_1_, *β*_2_ and *β*_12_ are regression coefficients calculated from experimental data; and *x*_1_ and *x*_2_ correspond to independent variables.

#### 4.3.2. Extraction by Conventional Agitation

Conventional agitation was carried out following the method previously described by Charles-Rodríguez [17]. Initially, 11.5 g of dried sample was placed in a flask containing 125 mL of water and a hydroalcohol solution (50:50) and extracted at 150 rpm for 24 h at room temperature (Innova 44 Incubator, New Brunswick Scientific Co., Inc., Edison, NJ, USA). The aqueous and hydroalcohol extracts were concentrated and stored as previously described for samples obtained by OH extraction (Section 4.3.1).

### 4.4. Analytical Methods

#### 4.4.1. Determination of Extraction Yields

The yield percentage for each extraction was determined as follows:

(2) $$Yield\;\left(\%\right)=\left(\frac{M_{EC}}{M_{ES}}\right)\times 100$$

where $M_{EC}$ is the mass of extract obtained at the end of the extraction process and $M_{ES}$ is the initial mass of dried sample used for the test.

#### 4.4.2. Total Phenolic Content (TPC)

The TPC was made by FC method adapted to microplate [38]. Initially, 5 µL of diluted sample (20 mg of extract in 5 mL of its respective solvent, water and a hydroalcohol solution) were placed in a 96-well microplate; 60 µL of FC reagent was then added and mixed for 2 min, followed by the addition of 15 µL of Na_2_CO_3_ solution (7.5% *w*/*v*) and 200 µL of distilled water. Finally, the reaction mixture was incubated at 60 °C for 5 min and the absorbance was measured at 750 nm in a fully automatic microplate lector (BIOBASE-EL 10A, Jinan, SHG, China). The values were compared with a calibration curve of GA at 0.05, 0.1, 0.2, 0.3, 0.4, 0.5, and 0.6 mg/L (*R*^2^ = 0.9973). The results were expressed as mg equivalents of GA per gram of extract. All experiments were made in triplicate.

#### 4.4.3. Phenolic Profile by Ultra High-Performance Liquid Chromatography (UHPLC)

It is crucial to perform a chromatographic analysis for the correct elucidation of the phenol composition as the presence of compounds in the extracts, such as reducing sugars (glucose and fructose), vitamin C, among others, can interfere with the accuracy of the Folin–Ciocalteu method [43]. The identification and quantification of phenolic compounds in the extracts were evaluated using Shimatzu Nexpera X2 equipment coupled with a diode array detector (Shimadzu, SPD-M20A, Tokyo, Japan). For this purpose, a reversed-phase Acquity UPLC BEH C18 column of 2.1 mm × 100 mm, 1.7 µm (Waters) and a precolumn filled with the same material. The temperature and the flow rate were 40 °C and 0.4 mL/min, respectively. The elution gradient used was in accordance with the previous report [44], where solvent A was a water/formic acid (0.1%) and acetonitrile as solvent B. For solvent B, the elution gradient was as follows: from 0.0 to 5.5 min eluent B at 5%, from 5.5 to 17 min was linearly increasing from 5 to 60%, from 17.0 to 18.5 min a linear increase to 100%; and, lastly, from 18.5 to 30.0 min, the column was equilibrated at 5%. The identification was conducted by comparing the ultraviolet spectra and retention times of samples with those of the standards. For each compound, calibration curves were conducted with concentrations between 2.5 and 250 mg/mL (*R*^2^ > 0.99); the compounds were identified and quantified at wavelengths between 209 and 370 nm. All measurements were performed in triplicate.

### 4.5. Bioactivity of Extracts

#### 4.5.1. Scavenging Properties

##### 2,2-Diphenyl-1-picrylhydrazyl (DPPH) Radical Scavenging Assay

The capacity of free radical capture of extracts was determined by the DPPH assay [17]. The DPPH microplate-adapted assay was conducted using 25 µL of each diluted sample at 20 to 2500 mg/L, dissolved in its respective extraction solvent, then the samples were mixed with a 200 µL of DPPH solution (150 µM, dissolved in absolute ethanol) in a 96-well microplate. The reaction was incubated under dark conditions at room temperature for 30 min. The absorbance was measured at 520 nm in a fully automatic microplate lector (BIOBASE-EL 10A, Jinan, SHG, China), using absolute ethanol as a control. The scavenging activity was expressed as DPPH radical scavenging activity percentage *(% RSA*), determined using the following equation:

(3) $$RSA\;\left(\%\right)=\left(\frac{A_{control}-A_{sample}}{A_{control}}\right)\times 100$$

where *A_contro_*_l_= control absorbance and A_sample_= sample absorbance. The scavenging activity was reported as EC_50_ (half-effective concentration) values, which expresses the concentration that gives the 50% maximal response of radical scavenging activity and was calculated from the regression equation given by the concentration–%RSA curve. All measurements were performed in triplicate.

##### 2,2′-Azino-di-[3-ethylbenzthiazoline Sulfonate] (ABTS) Radical Scavenging Assay

The scavenging capacity by the cation radical discoloration test (ABTS) of the extracts was conducted by the microplate-adapted assay as described by Jasso de Rodríguez [38] with minor modifications. The solution of ABTS was prepared at concentration of 7 mM in distilled water and mixed with a solution of potassium persulfate (2.45 mM); the mixture was kept at 4 °C during 14–16 h under dark conditions to ensure a stable oxidative state. The work solution was adjusted with ethanol at 20% to an absorbance of 0.700 ± 0.010 at 750 nm. In order to determine the scavenging activity, 10 µL of diluted samples were added to a 96-well microplate and mixed with 200 µL of work solution of ABTS, the reaction mixture was maintained for 10 min under dark conditions, and then the absorbance was measured at 750 nm, using the respective solvent as a control (i.e., water and a hydroalcohol solution). The results were calculated using the same Equation (3) described for the DPPH assay. The results were expressed as EC_50_ values. All experiments were carried out in triplicate.

#### 4.5.2. Reducing Properties

Ferric Reducing Antioxidant Power (FRAP) Assay

The FRAP assay involves the ability of extract to reduce ferric ions (Fe^3+^) [45]; the ferric reducing ability was evaluated according to the microplate-adapted methodology described by Lopéz-Romero [36]. The working solution of FRAP was made mixing 10 volumes of 300 mM acetate buffer (pH 3.6), 1 volume of 20 mM aqueous ferric chloride, and 1 volume of 40 mM TPTZ (HCl 40 mM as solvent). To evaluate the reducing activity, 280 µL of working solution was mixed with 20 µL of the extracts (0.5 mg/mL) in a 96-well microplate and put under dark conditions for 30 min to complete the reaction. The absorbance was read at 630 nm in a microplate reader (BMG Labtech, Ortenberg, Germany). Results were reported as µM Fe(ll)/g extract. All experiments were carried out in triplicate.

#### 4.5.3. Cell Viability Assay

Cell lines HeLa (human cervix carcinoma) and ARPE-19 (human retinal pigmented epithelium) were obtained from the American Type Culture Collection (ATCC; Rockville, MD, USA). Cells were cultured in DMEM supplemented with 5% FBS (Sigma, St. Louis, MO, USA). The evaluation of the cell viability was conducted by the MTT assay against ARPE-19 and HeLa cell line cultures, where metabolically active cells reduce the tetrazolium salt to colored formazan crystals and the amount of formazan produced is directly proportional to the number of viable cells [46]. The procedure was made according to Hernandez [47]. Initially, 50 µL (at 1 × 10^4^ cells) was incubated in a 96-well microplate for 24 h at 37 °C, with a 5% CO_2_ atmosphere. Then, 50 µL of medium containing different concentrations of extracts (previously dissolved in DMSO) were added and incubated for 48 h under the same conditions. Each well was washed with a PBS solution and refilled with fresh culture medium in the last 4 h. Finally, 10 µL of an MTT solution (5 mg/mL) was added to each well of the 96-well microplate and read at 570 and 650 nm in a microplate reader (iMark, Bio-Rad Laboratories, D.F., Mexico). The results were expressed as IC_50_ values that corresponded to the required concentration to inhibit 50% of the viable cells proliferation. All experiments were carried out in triplicate.

#### 4.5.4. Antifungal Activity in vitro

The antifungal activity was made by a microdilution technique according to a previous report [48], with some modifications. Briefly, extracts were diluted with 100 µL of sterile PDB to obtain different doses and were added into a sterile 96-well microplate. Then, 100 µL of the spore’s suspension of *R. stolonifer* or *F. oxysporum* at a concentration of 10^4^ spores/mL, was added. Fungal sporulation was monitored by changes in the optical density (OD) in fully automatic microplate lector (BIOBASE-EL 10A, Jinan, SHG, China) at 530 nm during 36 h (12 h intervals, at an incubation temperature of 25 ± 2 °C). A positive control was prepared by mixing 100 µL of sterile PDB with 100 µL of spore suspension. The percentage of growth inhibition (%) was calculated by the following equation:

(4) $$Inhibition\;\left(\%\right)=\left(\frac{OD_{control}-OD_{sample}}{OD_{control}}\right)\times 100$$

where $OD_{sample}$ represents the optical density of each treatment and $OD_{control}$ represents the optical density of the control. All treatments were replicated four times. The inhibition results were used to estimate the minimum inhibitory concentration (MIC) of extract that causes a 50% and 90% of reduction in fungal growth (MIC_50_ and MIC_90_, respectively).

### 4.6. Statistical Analysis

Statistical analyses of the data were performed by one-way analyses of variance (ANOVA) to detect significant differences and the Tukey mean comparison test (*p* < 0.05) using Minitab software version 17.0 (State College, PA, USA). The Probit analysis (SAS Program Version 9.1) was used to calculate the minimum inhibitory concentration of extract causing a 50% and 90% (MIC_50_ and MIC_90_, respectively) of reduction in fungal growth at *p* < 0.05 significant level.

## 5. Conclusions

The conditions and type of extraction technique can influence the properties and nature of compounds isolated from plant extracts. In this work, the phenolic composition of Rm and Mg fruit extracts was characterized by the presence of phenolic acids and flavonoids, showing that hydroalcohol extracts of both fruits exhibited the highest scavenging and reducing properties and antifungal activity. Among these, the RmH-C and MgH-C extracts are highlighted for their antifungal efficacy to inhibit the growth of *R. stolonifer* and *F. oxysporum*, respectively. Additionally, low cytotoxicity was observed against HeLa line cell in some treatments (RmH-C, RmH-OH5, and RmH-H10) while there was no cytotoxic effect for noncancerous ARPE-19 cell line.

The use of plants as a source of BCs is an area of relevance for the development of novel products to meet regional needs, and it is necessary to choose the appropriate extraction technique to guarantee their properties. This study reveals the potential of Rm and Mg fruits as novel sources of BCs for future applications in various areas, such as agrifood and pharmaceutical industries.

## Figures and Tables

**Table 1:** Yields (%) and total phenolic content (TPC) of *R. microphylla* (Rm) and *M. geometrizans* (Mg) fruit extracts obtained by conventional agitation (C) and ohmic heating (OH).

Extract	Yield (%)	TPC (mg GA/g Extract)
*Rm fruits*		
RmA-C	27.63 ( ± 1.12) ^b^	75.34 ( ± 6.48) ^a^
RmA-OH5	23.16 ( ± 1.66) ^b,c^	25.89 ( ± 1.00) ^c^
RmA-OH10	20.73 ( ± 1.55) ^c^	25.16 ( ± 2.35) ^c^
RmH-C	37.03 ( ± 1.99) ^a^	62.00 ( ± 3.34) ^a^
RmH-OH5	7.99 ( ± 1.95) ^d^	21.78 ( ± 2.49) ^c^
RmH-OH10	10.42 ( ± 0.35) ^d^	41.37 ( ± 4.25) ^b^
*Mg fruits*		
MgA-C	37.63 ( ± 3.52) ^a^	8.45 ( ± 1.04) ^a,b^
MgA-OH5	20.82 ( ± 1.35) ^b^	6.89 ( ± 0.74) ^b^
MgA-OH10	40.07 ( ± 1.28) ^a^	9.66 ( ± 1.92) ^a,b^
MgH-C	43.96 ( ± 5.93) ^a^	13.69 ( ± 4.88) ^a^
MgH-OH5	18.14 ( ± 1.90) ^b^	9.07 ( ± 1.07) ^a,b^
MgH-OH10	20.28 ( ± 3.76) ^b^	5.46 ( ± 0.82) ^b^

Values are presented as mean (± standard deviation, *n =* 3), different lowercase letters in the same column indicate statistically significant differences (*p* < 0.05) for each plant. A: aqueous extract; H: hydroalcohol extract; C: conventional agitation; OH: ohmic heating; 5 and 10: correspond to operating time (min) for OH extraction.

**Table 2:** Linear models describing the response variation of yields and total phenolic content (TPC) in function of time (A) and solvent (B) tested in experimental models for OH extraction, and their correspondent *R*^2^ coefficients.

Model	Response	Model Regression Equation	*R* ^2^
Rm fruits	Yields	15.51 + 0.001(A) – 10.01(B) + 0.486(A*B)	0.97
	TPC	14.41 + 1.886(A) – 12.21(B) + 2.032(A*B)	0.92
Mg fruits	Yields	8.80 + 2.137(A) + 7.20(B) – 1.710(A*B)	0.96
	TPC	8.40 – 0.084(A) + 4.28(B) – 0.638(A*B)	0.74

Rm: *Rhus microphylla*, Mg: *Myrtillocactus geometrizans*.

**Table 3:** Phenolic profile obtained by UHPLC of the different extracts of *R. microphylla* (Rm) fruits.

Compound	RT (min)	Wavelength (nm)	Extract (mg/L)					
			RmA-C^1^	RmA-OH5	RmA-OH10	RmH-C^1^	RmH-OH5	RmH-OH10
Gallic acid	2.21	280	203.20 ( ± 0.70) ^a^	9.18 ( ± 0.64) ^e^	12.83 ( ± 1.07) ^d^	98.60 ( ± 4.40) ^b^	16.21 ( ± 0.40) ^c^	14.69 ( ± 0.22) ^c,d^
p-cumaric acid+epicatechin	11.54	280	7.40 ( ± 0.20) ^b^	n.d.	n.d.	78.20 ( ± 1.50) ^a^	n.d.	n.d.
Catechin	7.15	280	n.d.	n.d.	n.d.	10.40 ( ± 0.40)	n.d.	n.d.
Ellagic acid	12.75	250	n.d.	8.73 ( ± 0.11) ^b^	8.80 ( ± 0.18) ^b^	2.90 ( ± 0.10) ^c^	9.43 ( ± 0.23) ^a^	9.59 ( ± 0.02) ^a^
Ferulic acid	13.02	320	5.70 ( ± 0.10) ^a^	n.d.	n.d.	6.10 ( ± 1.30) ^a^	n.d.	n.d.
Resveratrol	14.48	308	n.d.	n.d.	n.d.	2.90 ( ± 0.01)	n.d.	n.d.

Values are presented as mean (± standard deviation, *n =* 3), different lowercase letters in the same row indicate statistically significant differences (*p* < 0.05). A: aqueous extract; H: hydroalcohol extract; C: conventional agitation; OH: ohmic heating; 5 and 10: correspond to operating time (min) for OH extraction. n.d.: not detected. RT: retention time. ^1^﻿Adapted from Charles-Rodríguez [17].

**Table 4:** Phenolic profile obtained by UHPLC of the different extracts of *M. geometrizans* (Mg) fruits.

Compound	RT (min)	Wavelength (nm)	Extract (mg/L)					
			MgA-C	MgA-OH5	MgA-OH10	MgH-C	MgH-OH5	MgH-OH10
Rosmarinic acid	12.22	329	n.d.	n.d.	n.d.	12.36 (± 0.01)	n.d.	n.d.
Ellagic acid	12.75	250	4.76 (± 0.14) ^c^	8.47 (± 0.03)^a^	n.d.	5.12 (± 0.01) ^b^	8.62 (± 0.03) ^a^	8.59 (± 0.01) ^a^
Ferulic acid	13.02	320	8.30 (± 0.02) ^a^	n.d.	n.d.	8.30 (± 0.00) ^a^	n.d.	n.d.
o-cumaric acid	13.60	280	3.10 (± 0.01) ^a^	n.d.	n.d.	3.15 (± 0.10) ^a^	n.d.	n.d.
Rutin	12.74	350	n.d.	n.d.	n.d.	2.76 (± 0.07) ^a^	n.d.	n.d.

Values are presented as mean (± standard deviation, *n =* 3), different lowercase letters in the same row indicate statistically significant differences (*p* < 0.05). A: aqueous extract; H: hydroalcohol extract; C: conventional agitation; OH: ohmic heating; 5 and 10: correspond to operating time (min) for OH extraction. n.d.: not detected. RT: retention time.

**Table 5:** Scavenging (DPPH, ABTS) and reducing (FRAP) properties of *R. microphylla* (Rm) and *M. geometrizans* (Mg) fruit extracts obtained by conventional agitation (C) and ohmic heating (OH).

Extract	DPPH EC_50_ (mg/mL)	ABTS EC_50_ (mg/mL)	FRAP µM Fe(II)/g extract
*Rm fruit*			
RmA-C	0.36 (± 0.02) ^a^	0.17 (± 0.01) ^a^	1662.00 (± 108.30) ^a^
RmA-OH5	0.94 (± 0.03) ^d^	0.48 (± 0.03) ^b^	840.23 (± 60.65) ^b^
RmA-OH10	0.72 (± 0.09) ^c^	0.41 (± 0.02) ^b^	660.52 (± 12.70) ^c^
RmH-C	0.32 (± 0.04) ^a^	0.24 (± 0.02) ^a^	1589.39 (± 53.02) ^a^
RmH-OH5	0.60 (± 0.02) ^b,c^	0.20 (± 0.11) ^a^	840.50 (± 23.03) ^b^
RmH-OH10	0.56 (± 0.04) ^b^	0.21 (± 0.00) ^a^	485.51 (± 13.92) ^d^
*Mg fruit*			
MgA-C	8.75 (± 0.92) ^b^	5.65 (± 0.87) ^b,c^	136.94 (± 4.92) ^d^
MgA-OH5	21.06 (± 2.59) ^c^	6.95 (± 1.19) ^c^	168.18 (± 1.95) ^c^
MgA-OH10	9.62 (± 1.34) ^b^	6.19 (± 0.73) ^b,c^	173.06 (± 5.73) ^c^
MgH-C	5.25 (± 0.86) ^a^	3.79 (± 0.37) ^a^	255.78 (± 24.36) ^a^
MgH-OH5	17.92 (± 0.87) ^c^	7.04 (± 0.61) ^c^	207.56 (± 10.65) ^b^
MgH-OH10	16.76 (± 3.62) ^c^	4.59 (± 0.31) ^b^	174.14 (± 6.57) ^c^

Values are presented as mean (± standard deviation, *n =* 3), different lowercase letters in the same column indicate statistically significant differences (*p* < 0.05) for each plant. A: aqueous extract; H: hydroalcohol extract; C: conventional agitation; OH: ohmic heating; 5 and 10: correspond to operating time (min) for OH extraction.

**Table 6:** Antiproliferative activity of *R. microphylla* (Rm) and *M. geometrizans* (Mg) fruit extracts obtained by conventional agitation (C) and ohmic heating (OH) in ARPE-19 and HeLa cell lines.

Extract	Cell lines IC_50_ (µg/mL)	
	ARPE-19	HeLa
*Rm fruit*		
RmA-C	> 800 ^a^	> 800 ^c^
RmA-OH5	> 800 ^a^	> 800 ^c^
RmA-OH10	> 800 ^a^	> 800 ^c^
RmH-C	> 800 ^a^	417.73 (± 29.06) ^a^
RmH-OH5	> 800 ^a^	705.73 (± 21.59) ^b^
RmH-OH10	> 800 ^a^	615.33 (± 64.56) ^b^
*Mg fruit*		
MgA-C	> 800 ^a^	> 800 ^c^
MgA-OH5	> 800 ^a^	> 800 ^c^
MgA-OH10	> 800 ^a^	> 800 ^c^
MgH-C	> 800 ^a^	> 800 ^c^
MgH-OH5	> 800 ^a^	> 800 ^c^
MgH-OH10	> 800 ^a^	> 800 ^c^

IC_50_ values represent a mean and standard deviation (± SD; *n =* 3) of three independent experiments. Different lowercase letters in the same column indicate statistically significant differences (*p* < 0.05). A: aqueous extract; H: hydroalcohol extract; C: conventional agitation; OH: ohmic heating; 5 and 10: correspond to operating time (min) for OH extraction.

**Table 7:** Minimum inhibitory concentrations (MICs) of *R. microphylla* (Rm) and *M. geometrizans* (Mg) fruit extracts causing a reduction of 50 and 90% of mycelia growth of *R. stolonifer* and *F. oxysporum*.

Extract	MIC_50_ (mg/L)	95% Fiducial Limits		MIC_90_ (mg/L)	95% Fiducial Limits	
		Lower	Upper		Lower	Upper
*R. stolonifer*						
RmH-C	1599	1291	1784	2219	1974	2927
RmH-OH5	2366	2164	2541	4432	3825	5819
RmH-OH10	2918	n.d.	n.d.	40912	n.d.	n.d.
MgH-C	8415	7355	10026	54621	37318	92963
*F. oxysporum*						
RmH-C	5940	4551	12395	73199	24989	1653990
MgH-C	1915	1631	2256	4881	3908	6661

A: aqueous extract; H: hydroalcohol extract; C: conventional agitation; OH: ohmic heating; 5 and 10: correspond to operating time (min) for OH extraction. The MICs values of extracts with low inhibition percentages ( < 40%) were not included. n.d.= not detected.

**Table 8:** Factorial design for ohmic heating (OH) extraction.

Run	Solvent	Time (min)
1	Water	5
2	Water	10
3	Hydroalcohol solution (50:50)	5
4	Hydroalcohol solution (50:50)	10

## Data Availability

Not applicable.
